# Prognostic Value of the Level of Urine Dipstick Proteinuria in Gastric Cancer in the Korean Population

**DOI:** 10.3390/cancers17172743

**Published:** 2025-08-23

**Authors:** Yeo Ju Sohn, Sol Lee, Junghwa Kim, Insun Ryou, Eunjin Jeong, Jae-Hong Ryoo, Hyejin Chun

**Affiliations:** 1 Department of Family Medicine, Ewha Women’s University Seoul Hospital, Ewha Women’s University College of Medicine, Seoul 07804, Republic of Korea; yeojusohn@ewha.ac.kr (Y.J.S.); 01581s@eumc.ac.kr (J.K.); 01027s@ewha.ac.kr (I.R.); 01591s@eumc.ac.kr (E.J.); 2 Department of Family Medicine, Ewha Women’s University Mokdong Hospital, Ewha Women’s University College of Medicine, Seoul 07985, Republic of Korea; 01473@eumc.ac.kr; 3 Departments of Occupational and Environmental Medicine, School of Medicine, Kyung Hee University, Seoul 02447, Republic of Korea; armani131@khu.ac.kr

**Keywords:** urine dipstick test, proteinuria, gastric cancer, risk stratification, cohort study

## Abstract

Background/Objectives: Proteinuria, a marker of renal dysfunction, has been implicated in cancer risk, yet its role in gastric carcinogenesis remains underexplored in high-incidence populations. This study evaluated the association between urine dipstick proteinuria severity and gastric cancer incidence in a nationwide Korean cohort. Methods: We analyzed data from the Korean National Health Insurance Service–National Sample Cohort, including 220,941 adults aged > 40 years, without a diagnosis of cancer, who received health examinations in 2009. Proteinuria was classified by single dipstick testing as negative, 1+, or ≥2+. Participants were followed for a mean of 4.37 ± 0.49 years (965,601.2 person-years). Multivariable Cox proportional hazards models adjusted for age, sex, body mass index, systolic blood pressure, fasting glucose, LDL cholesterol, estimated glomerular filtration rate, smoking status, alcohol intake, and physical activity were used to estimate hazard ratios (HRs) and 95% confidence intervals (CIs). Results: During follow-up, 1934 participants (0.88%) developed gastric cancer. A significant dose–response relationship emerged (*p* for trend = 0.037). In fully adjusted models, 1+ proteinuria conferred no significant risk increase (HR 1.10; 95% CI, 0.80–1.51), whereas ≥2+ proteinuria was associated with a 42% higher gastric cancer risk (HR 1.42; 95% CI, 1.00–2.02). Conclusions: Severe dipstick proteinuria independently predicts elevated gastric cancer risk in Korean adults. Integration of urine dipstick testing into gastric cancer screening protocols may offer a simple, cost-effective strategy for risk stratification, particularly in high-incidence settings.

## Simple Summary

Detecting protein in urine with a dipstick test is a routine, low-cost procedure typically used to identify kidney problems. Emerging evidence suggests that proteinuria may also reflect systemic processes linked to cancer development. In this retrospective cohort of 220,941 Korean adults aged 40 years or older who underwent health examinations in 2009, we investigated whether dipstick-measured proteinuria predicts subsequent gastric cancer risk over a mean follow-up of 4.37 years. Compared with individuals without proteinuria, those with high-grade proteinuria (≥2+) exhibited a 42% greater risk of developing gastric cancer. Incorporating urine dipstick testing into existing screening paradigms could enable earlier identification of high-risk individuals and enhance prevention efforts in regions with elevated gastric cancer incidence.

## 1. Introduction

Gastric cancer constitutes a major global health burden, with approximately 1.1 million new cases diagnosed worldwide in 2020, accounting for 5.6% of all cancer incidences and ranking as the fourth leading cause of cancer-related mortality [1,2]. The burden is particularly pronounced in Asia, especially East Asia, where the age-standardized incidence rate reaches 22.4 per 100,000 population, significantly exceeding the global average of 11.1 per 100,000 [3].

In South Korea, a total of 343,895 patients who were diagnosed with gastric cancer between 1 January 1999 and 31 December 2021 were alive as of 1 January 2022 [4]. South Korea’s National Cancer Screening Program, launched in 1999, has markedly improved gastric cancer outcomes [5]. The program has led to high rates of early detection; 66.0% of gastric cancer cases were diagnosed at the localized stage and 18.6% at the regional stage in 2021. Furthermore, the program has contributed to a marked increase in the 5-year relative survival rate, which reached 77.9% for the period 2017–2021. The age-standardized mortality rate has continued its downward trend, decreasing to 5.9 per 100,000 in 2021 [6].

Recent investigations have revealed a significant association between chronic kidney disease (CKD) and increased cancer risk. Patients with CKD demonstrate up to 108% higher incidence of overall cancer incidence compared to individuals without CKD, particularly those with moderate CKD (eGFR < 60 mL/min/1.73 m^2^) [7]. A large-scale Korean cohort study of pre-dialytic CKD patients confirmed a significantly elevated risk of digestive cancers, with a standardized incidence ratio (SIR) of 1.25 for gastric cancer. Furthermore, gastric cancer patients with CKD exhibit higher postoperative complication rates and reduced long-term survival compared to those with normal kidney function [8].

Proteinuria, traditionally recognized as a marker of renal damage, has recently emerged as a potential indicator of systemic inflammatory responses and vascular endothelial dysfunction, extending beyond simple kidney impairment. A comprehensive analysis using the Korean National Health Insurance Service database, which included 9,714,387 subjects, demonstrated that overt proteinuria was associated with a 15.4% increased risk of overall cancer development (adjusted HR 1.154, 95% CI 1.134–1.173), with the risk of cancer incidence rising proportionally according to proteinuria changes over a four-year period [9].

Given Korea’s high incidence of gastric cancer and its well-established national screening program, this study was conducted to examine whether semi-quantitative proteinuria, as assessed by routine urine dipstick testing, exhibits a dose–response relationship with gastric cancer risk. The goal was to evaluate the potential of proteinuria as a noninvasive, cost-effective biomarker for targeted screening.

## 2. Materials and Methods

The national health insurance system in South Korea covers over 97% of the population, indicating that its database can reliably represent the medical service utilization of the entire Korean population [10]. Since 2009, Korean adults aged ≥40 years have been required to undergo biennial health examinations, with results that are stored by the National Health Insurance Service (NHIS). For research purposes, NHIS provides a de-identified sample cohort linked to cancer registry data from Statistics Korea. The study protocol was approved by the Institutional Review Board of Kyung Hee University Hospital, which waived the requirement for informed consent due to retrospective analysis of anonymized records.

A total of 223,551 individuals who underwent a medical health checkup in 2009 were included in the National Health Information Database. Of these, 1846 individuals with a prior diagnosis of gastric cancer (ICD code C16) between 2002 and the date of the 2009 examination were excluded. An additional 772 individuals were excluded due to missing proteinuria data. Because two participants had more than one exclusion criterion, 220,941 participants were included in the final analysis and were followed for the incidence of gastric cancer. The total follow-up period was 965,601.2 person-years, with a mean follow-up period of 4.37 years (standard deviation [SD], 0.49).

We conducted a retrospective cohort study of NHIS health-check examinees aged ≥40 years between 2009 and 2019, including only those with available urine dipstick proteinuria results.

The exclusion criteria were as follows:

- History of gastric cancer (ICD-10 code C16) before baseline health examination;
- Diagnosis of any other malignancy during the follow-up period;
- End-stage renal disease (KDIGO stage 5) or undergoing dialysis;
- Prior treatment for proteinuria (e.g., renin–angiotensin system inhibitors).

After excluding 1846 individuals with preexisting gastric cancer and 772 individuals with missing proteinuria data, a total of 220,941 participants were included in the final analysis. The total follow-up was 965,601.2 person-years, with a mean follow-up of 4.37 ± 0.49 years.

The general health check-up provided by the NHIC was conducted in 2 stages. The first stage involved large-scale screening designed to identify potential diseases in the asymptomatic population. The second stage comprised follow-up consultations and detailed assessments to confirm disease diagnosis. These examinations included questionnaires addressing lifestyle factors and medical history. Study data encompassed physical activity parameters, lifestyle questionnaire responses, anthropometric measurements, and laboratory test results. Smoking exposure was quantified in pack-years based on smoking-related questionnaire responses. Alcohol intake was defined as consumption exceeding three times per week. Physical activity was defined as either moderate-intensity exercise for ≥30 min daily on >4 days per week, or vigorous-intensity exercise for ≥20 min daily on >4 days per week. Body mass index (BMI) was calculated as weight (kg) divided by height squared (m^2^). Blood pressure was measured by trained examiners.

Laboratory variables assessed during health examinations included: fasting blood glucose, total cholesterol, triglycerides, high-density lipoprotein (HDL) cholesterol, low-density lipoprotein (LDL) cholesterol, serum creatinine (SCr), aspartate aminotransferase (AST), alanine aminotransferase (ALT), and γ-glutamyltransferase (GGT). Renal function was evaluated using estimated glomerular filtration rate (eGFR), calculated via the Chronic Kidney Disease Epidemiology Collaboration (CKD-EPI) equation: eGFR = 141 × min (SCr/K, 1)^a^ × max (SCr/K, 1)^−1.209^ × 0.993^age^ × 1.018 [if female] × 1.159 [if Black], where K equals 0.7 for females and 0.9 for males, α equals −0.329 for females and −0.411 for males, and min/max represent the minimum/maximum values of SCr/K or 1, respectively.

The National Health Insurance database was linked to the diagnosis of disease data provided by Statistics Korea. In this study, the baseline was defined as the date of the participant’s health check-up conducted in or after 2009, and the endpoint was the date of the diagnosis of gastric cancer if it was before 31 December 2013. The diagnosis of gastric cancer was defined as ICD code C16. The primary clinical outcome of interest was the outcome of gastric cancer.

Baseline demographics and clinical variables included age, sex, body mass index (BMI), systolic blood pressure (BP), fasting blood glucose, LDL cholesterol, and estimated glomerular filtration rate (eGFR; CKD-EPI equation). Lifestyle factors, including smoking (pack-years), alcohol intake (≥3 times per week), and physical activity (defined as ≥30 min of moderate-intensity ≥4 days per week or ≥20 min of vigorous-intensity ≥4 days per week), were assessed via self-administered questionnaires.

Data were expressed as means ± standard deviations (SD) or medians with interquartile ranges (IQR) for continuous variables, and as proportions (%) for categorical variables. One-way ANOVA and chi-square (χ^2^) tests were used to compare baseline characteristics of study participants across the three groups defined by urine dipstick protein levels. Person-years were calculated as the sum of follow-up times from the baseline to either the date of gastric cancer diagnosis or 31 December 2013, whichever came first.

To evaluate the association between urine dipstick protein levels and incident gastric cancer, we used Cox proportional hazards models to estimate adjusted hazard ratios (HRs) and 95% confidence intervals (CI) for incident gastric cancer comparing groups 2 and 3 with group 1 (negative proteinuria).

The models were adjusted for multiple potential confounders. In the multivariate models, we included variables that might confound the relationship between urine dipstick protein levels and incident gastric cancer, which include age, sex, BMI, systolic BP, fasting blood glucose, LDL cholesterol, eGFR, smoking amount (pack-years), alcohol intake, and physical activity.

To test the validity of the Cox proportional hazard models, we checked the proportional hazard assumption. The proportional hazard assumption for the Cox models was evaluated using log-minus-log survival plots and found not to be violated. *p*-values < 0.05 were considered statistically significant. All statistical analyses were performed using SAS (version 9.4, SAS Institute Inc., Cary, NC, USA).

## 3. Results

During 965,601.2 person-years of follow-up, a total of 1934 incident cases of gastric cancer (0.88%) were identified between 2009 and 2013. The baseline characteristics of the study participants according to the three groups of urine dipstick protein levels are presented in Table 1. At baseline, the mean (SD) age and BMI of participants were 58.0 (8.8) years and 24.0 (2.9) kg/m^2^, respectively. Significant differences were observed among all listed variables across the three proteinuria groups, except for LDL cholesterol and physical activity.

Compared to participants without incident gastric cancer, those who developed gastric cancer were older (62.9 vs. 58.0 years) and were more likely to have a less favorable metabolic profile at baseline. As expected, all clinical variables showed statistically significant differences between the two groups except for BMI, HDL cholesterol, SCr, ALT and physical activity (Supplementary Table S1).

Table 2 shows the HRs and 95% CIs for gastric cancer across the three groups of urine dipstick protein levels. In the unadjusted model, the HRs (95% CIs) for gastric cancer were 1.10 (0.80–1.51) in group 2 and 1.68 (1.19–2.39) in group 3, compared to group 1 (negative proteinuria). The trend across groups was statistically significant (*p* for trend = 0.005).

These associations remained statistically significant, even after additional adjustments for potential confounders in a multivariate adjusted model, the adjusted HRs (95% CI) for gastric cancer were 0.92 (0.67–1.28) and 1.42 (1.00–2.02), respectively (*p* for trend = 0.037) (Table 3).

## 4. Discussion

This study represents a comprehensive retrospective cohort analysis of 220,941 Korean individuals, demonstrating a significant association between urine dipstick proteinuria and gastric cancer risk. Participants with proteinuria ≥2+ exhibited a 42% increased risk of gastric cancer development compared to those with negative proteinuria (adjusted HR 1.42, 95% CI 1.00–2.02), with a statistically significant dose-response trend (*p* for trend = 0.037). These findings provide additional evidence supporting the association between chronic kidney disease, including proteinuria, and increased gastric cancer risk.

The mechanistic association between proteinuria and gastric cancer development can be explained by dysregulation of the renin-angiotensin system (RAS). Chronic elevation of angiotensin II (Ang II) triggers a cascade of pathophysiological events that extend beyond conventional cardiovascular effects. RAS components, including angiotensin-converting enzyme (ACE), Ang II, and angiotensin receptors (AT1R and AT2R), are expressed in various cancer tissues and have been implicated in tumorigenesis [11]. Ang II/AT1R signaling promotes cancer progression through several mechanisms, such as enhancing the stimulation of angiogenesis via upregulation of vascular endothelial growth factor (VEGF), stimulating cellular proliferation through transforming growth factor-β (TGF-β) pathways, and facilitating metastasis. The overexpression of AT1R is typically associated with more aggressive tumor characteristics, including larger tumor size, higher histological grade, and increased vascular density [8]. In contrast, the Ang II/AT2R pathway generally exerts tumor-suppressive effects by promoting apoptosis and inhibiting cellular proliferation [12].

The inflammatory milieu resulting from chronic kidney dysfunction plays a significant role in gastric carcinogenesis. Persistent proteinuria induces a pro-inflammatory environment marked by elevated cytokines, such as interleukin-6 (IL-6), tumor necrosis factor-α (TNF-α), and interleukin-1β (IL-1β) [13]. These inflammatory mediators are pivotal in promoting gastric cancer development through activation of transcription factors, including nuclear factor-κB (NF-κB), signal transducer and activator of transcription 3 (STAT3), and activator protein-1 (AP-1) [14]. IL-6 emerges as a particularly important cytokine in this context, as it has been consistently associated with poor prognosis in patients with gastric cancer [15]. The chronic inflammatory environment activates pro-inflammatory pathways, leading to sustained cytokine production and the establishment of a self-perpetuating cycle that facilitates malignant transformation. Moreover, these inflammatory responses compromise immune surveillance, enabling malignant cells to circumvent immune recognition [16].

Proteinuria serves as a systemic marker of vascular endothelial dysfunction, a key contributor to cancer development and progression [17]. Endothelial dysfunction—characterized by impaired nitric oxide (NO) production, increased vascular permeability, and compromised immune cell trafficking—amplifies several hallmarks of malignancy, including sustained proliferative signaling, immune evasion, and enhanced angiogenesis [18,19]. The compromised endothelial barrier associated with proteinuria facilitates tumor cell intravasation and extravasation, thereby promoting metastatic dissemination [20]. Additionally, endothelial dysfunction influences the expression of immune checkpoint molecules, such as programmed death-ligand 1 (PD-L1), and promotes the secretion of immunosuppressive cytokines that inhibit cytotoxic T-cell infiltration and function [21]. These processes collectively contribute to the establishment of a tumor-supportive microenvironment that favors malignant progression while simultaneously compromising anti-tumor immune responses.

The association between proteinuria and gastric cancer can also be attributed to oxidative stress-mediated DNA damage. Chronic inflammation associated with kidney dysfunction leads to the generation of reactive oxygen species (ROS) that can directly damage DNA through the formation of oxidative adducts. 8-hydroxy-2′-deoxyguanosine (8-OHdG) is the most abundant oxidative DNA lesion and a widely recognized biomarker of oxidative stress with consistently elevated levels observed in gastric cancer tissues [21,22]. ROS-induced DNA damage activates the DNA damage response (DDR) pathway, which can either promote cellular repair or trigger apoptosis depending on the extent of damage [22]. However, chronic exposure to moderate oxidative stress can overwhelm repair mechanisms, leading to the accumulation of somatic mutations that drive gastric carcinogenesis [23]. Moreover, oxidative stress impairs the function of tumor suppressor gene products, particularly p53, which plays a central role in coordinating cellular responses to DNA damage. Under conditions of severe oxidative stress, p53 may paradoxically activate pro-oxidant pathways, further contributing to cellular dysfunction and malignant transformation [24].

The identification of proteinuria as a significant risk factor for gastric cancer has important implications for clinical practice, particularly in populations with high gastric cancer incidence, such as South Korea. The urine dipstick test is a simple, cost-effective, and widely available screening tool that could facilitate risk stratification for gastric cancer screening programs [25]. Given the proven efficacy of South Korea’s National Cancer Screening Program in reducing gastric cancer-related mortality, incorporating proteinuria assessment into routine health checkups could improve the early identification of high-risk individuals who might benefit from more intensive surveillance [26]. Notably, the dose-response relationship observed in this study suggests the need for particular attention to individuals with proteinuria of grade 2+ or higher, who may represent a subgroup at substantially elevated risk [27].

Beyond its role as a risk factor for gastric cancer development, proteinuria may also serve as a prognostic indicator in cancer patients. Studies in other malignancies have shown that proteinuria is associated with poorer clinical outcomes, including increased cancer-specific mortality and reduced overall survival [28]. In patients with colorectal cancer, for instance, proteinuria has been identified as an independent prognostic factor, particularly in early-stage disease [29]. This prognostic significance likely reflects the systemic nature of the underlying pathophysiological processes common to both proteinuria and cancer progression.

The mechanistic understanding of proteinuria-associated gastric cancer risk also presents potential therapeutic opportunities. Renin-angiotensin system inhibitors, including ACE inhibitors and angiotensin receptor blockers (ARBs), have demonstrated anti-tumor effects in preclinical models. Meta-analyses of clinical studies have suggested that the use of RAS inhibitors is associated with improved survival outcomes in cancer patients, with beneficial effects observed across multiple cancer types [30]. The proposed anti-tumor mechanisms of RAS inhibitors include suppression of angiotensin II-mediated tumor neovascularization, inhibition of cellular proliferation signaling pathways, and attenuation of metastatic potential.

Several methodological limitations must be considered when interpreting these findings. First, the use of a single urine dipstick measurement may not accurately reflect the chronicity of proteinuria, as urinary protein excretion can vary, depending on hydration status, physical activity, and acute illness. Second, the relatively short mean follow-up period of 4.37 years may be insufficient to fully observe the long-term development of gastric cancer, which typically occurs over decades. Additionally, the retrospective design of the study limits causal inference, making it difficult to determine whether proteinuria precedes the onset of gastric cancer or reflects an early manifestation of subclinical malignancy. Moreover, although our study did not perform subgroup analyses for major chronic systemic diseases characterized by proteinuria—such as diabetic nephropathy, lupus nephritis, or hypertensive nephrosclerosis—existing literature indicates that these conditions confer a significantly elevated malignancy risk through mechanisms of chronic inflammation, immune dysregulation, and prolonged exposure to immunosuppressive therapies. Future investigations should stratify proteinuria patterns by underlying disease etiology and assess their differential impact on cancer incidence to inform tailored, disease-specific prevention strategies.

The study’s observational design is susceptible to confounding by unmeasured variables that may influence both the development of proteinuria and the risk of gastric cancer. Important gastric cancer risk factors—such as *Helicobacter pylori* infection status, family history of gastric cancer, dietary factors, and specific lifestyle behaviors—were not comprehensively evaluated. Significantly, dietary factors including high salt intake, consumption of smoked or preserved foods, and low fruit and vegetable intake have been firmly established as gastric cancer risk factors, especially in East Asian populations. Moreover, overall nutritional status and specific dietary habits play a critical role in gastric carcinogenesis. In regions with a notably high incidence of gastric cancer, involvement of oncology-trained nutrition specialists has demonstrated improvements in both prognosis and prevention [31]. Anti-inflammatory, plant-based dietary patterns are associated with reduced gastric mucosal inflammation and sustained decreases in gastric cancer risk. Accordingly, our findings suggest that personalized nutritional counseling—tailored according to the dietary inflammatory index—for proteinuria individuals at high risk may interrupt the proteinuria–inflammation–neoplasia axis and thereby lower gastric cancer incidence. Finally, reverse causation cannot be fully excluded, since subclinical gastric malignancy itself might contribute to the onset of proteinuria.

Large-scale prospective cohort studies with extended follow-up periods are needed to clarify the temporal relationship between proteinuria and gastric cancer development. These studies should include repeated measurements of proteinuria to account for the dynamic nature of kidney function and its association with cancer risk. The identification of proteinuria as a gastric cancer risk factor also presents an opportunity to develop integrated biomarker panels that combine proteinuria assessment with established or emerging risk indicators.

The findings of this study have significant implications for population-based cancer screening strategies, particularly in regions with a high incidence of gastric cancer. Integrating proteinuria assessment into existing health screening programs may improve the identification of high-risk individuals without requiring substantial additional resources. Given the widespread availability, simplicity, and low cost of urine dipstick testing, this approach is feasible for implementation across diverse healthcare settings and could serve as a valuable adjunct to current screening practices.

## 5. Conclusions

This large-scale retrospective cohort study provides compelling evidence for a significant association between urine dipstick proteinuria and gastric cancer risk in the Korean population. The observed dose-response relationship and the magnitude of the association—42% increased risk for proteinuria grade 2+ or higher—support the potential clinical utility of proteinuria assessment in gastric cancer risk stratification. These findings carry important clinical implications, particularly in the context of population-based cancer screening programs. However, the implementation of proteinuria-based risk stratification strategies requires validation through prospective studies and careful evaluation of the potential benefits and harms associated with intensified screening. Integrating proteinuria assessment into existing gastric cancer screening efforts represents a promising approach to enhancing early detection and improving outcomes, especially in high-risk populations.

## Figures and Tables

**Table 1:** Baseline characteristics of participants according to the levels of proteinuria.

Characteristics	Overall	Proteinuria			
		Negative (−)	Mild (1+)	Moderate to Severe (≥2+)	*p* for Trend *
*n*	220,941	214,537	4146	2258	
Person-year (total)	965,601.2	938,086.1	17,926.3	9588.8	
Person-year (average)	4.37 ± 0.5	4.37 ± 0.5	4.32 ± 0.6	4.24 ± 0.7	<0.001
Age (years)	58.0 ± 8.8	58.0 ± 8.7	59.7 ± 9.3	60.3 ± 9.4	<0.001
Sex (men)	124,003 (56.1)	120,131 (56.0)	2463 (59.4)	1409 (62.4)	<0.001
BMI (kg/m^2^)	24.0 ± 2.9	24.0 ± 2.9	24.5 ± 3.3	24.8 ± 3.4	<0.001
Smoking amount (pack-year)	7.78 ± 13.9	7.7 ± 13.8	9.3 ± 15.8	9.6 ± 16.1	<0.001
Alcohol intake (%)	31,595 (14.3)	30,679 (14.3)	705 (17.0)	397 (17.6)	<0.001
Physical activity (%)	37,339 (16.9)	36,257 (16.9)	713 (17.2)	386 (17.1)	0.657
Systolic BP (mmHg)	125.3 ± 15.2	125.1 ± 15.1	128.9 ± 16.7	131.2 ± 17.5	<0.001
Diastolic BP (mmHg)	77.7 ± 9.9	77.6 ± 9.9	79.6 ± 10.7	80.1 ± 11.3	<0.001
Total cholesterol (mg/dL)	200.1 ± 37.5	200.0 ± 37.3	201.6 ± 40.7	203.8 ± 45.1	<0.001
Triglyceride (mg/dL)	118 (83–171)	118 (83–170)	130 (90–192)	138 (95–205)	<0.001
HDL cholesterol (mg/dL)	55.4 ± 32.4	55.5 ± 32.5	53.6 ± 24.2	52.9 ± 25.7	<0.001
LDL cholesterol (mg/dL)	118.4 ± 39.1	118.4 ± 39.0	117.2 ± 40.1	117.8 ± 44.2	0.492
Fasting glucose (mg/dL)	100.8 ± 25.4	110.4 ± 24.7	111.9 ± 38.7	118.0 ± 46.1	<0.001
Creatinine (mg/dL)	1.2 ± 1.5	1.1 ± 1.45	1.3 ± 1.87	1.5 ± 2.91	<0.001
eGFR (mL/min/1.73 m^2^)	80.7 ± 20.2	80.9 ± 20.0	75.2 ± 22.6	70.1 ± 24.6	<0.001
Development of gastric cancer (%)	1934 (0.9)	1863 (0.9)	39 (0.9)	32 (1.4)	0.010

* *p*-value by ANOVA test for continuous variables and Chi-square test for categorical variables.

**Table 2:** Baseline characteristics of participants according to the incidence of gastric cancer.

Characteristic	Incident Gastric Cancer		
	No	Yes	*p*-Value *
*n*	219,007	1934	
Age (years)	58.0 ± 8.7	62.9 ± 9.5	<0.001
Sex (men)	122,574 (56.0)	1429 (73.9)	<0.001
BMI (kg/m^2^)	24.0 ± 2.9	23.9 ± 2.9	0.240
Smoking amount (pack-year)	7.7 ± 13.8	11.5 ± 16.1	<0.001
Alcohol intake (%)	31,318 (14.3)	395 (20.4)	<0.001
Physical activity (%)	37,012 (16.9)	354 (18.3)	0.112
Systolic BP (mmHg)	125.3 ± 15.2	127.9 ± 15.6	<0.001
Diastolic BP (mmHg)	77.7 ± 9.9	78.6 ± 10.2	<0.001
Total cholesterol (mg/dL)	200.1 ± 37.4	196.7 ± 41.1	<0.001
Triglyceride (mg/dL)	141.7 ± 93.8	146.1 ± 95.3	0.044
HDL cholesterol (mg/dL)	55.4 ± 32.2	54.7 ± 39.4	0.460
LDL cholesterol (mg/dL)	118.4 ± 39.0	115.7 ± 39.3	0.002
Fasting glucose (mg/dL)	100.8 ± 25.4	103.4 ± 27.3	<0.001
Creatinine (mg/dL)	1.15 ± 1.48	1.21 ± 1.76	0.145
eGFR (mL/min/1.73 m^2^)	80.7 ± 20.2	78.1 ± 19.4	<0.001
Proteinuria level			0.010
Negative (−)	212,656 (97.1)	1863 (96.3)	
Mild (1+)	4161 (1.9)	39 (2.0)	
Moderate to Severe (≥2+)	2190 (1.0)	32 (1.7)	

* *p*-value by *t*-test for continuous variables and Chi-square test for categorical variables.

**Table 3:** Hazard ratios (HRs) and 95% confidence intervals (CI) for the incidence of gastric cancer according to the three groups of urine protein level.

Proteinuria	Person-Year	Incidence Cases	Incidence Density (per 10,000 Person-Year)	HR (95% CI)	
				Model 1	Model 2
Negative (−)	938,086.1	1863	19.8	1.00 (reference)	1.00 (reference)
Mild (1+)	17,926.3	39	21.7	1.10 (0.80–1.51)	0.92 (0.67–1.28)
Moderate to Severe (≥2+)	9588.8	32	33.4	1.68 (1.19–2.39)	1.42 (1.00–2.02)
*p* for trend				0.005	0.037

Model 2 was adjusted for age, gender, BMI, systolic BP, fasting blood glucose, LDL cholesterol, eGFR, smoking amount (pack-year), alcohol intake, and physical activity.

## Data Availability

The original contributions presented in this study are included in the article/supplementary material. Further inquiries can be directed to the corresponding author.

## Supplementary Materials

The following supporting information can be downloaded at: https://www.mdpi.com/article/10.3390/cancers17172743/s1, Table S1: Comparison between participants with and without incident gastric cancer.

## Abbreviations

NHIC	National Health Insurance Corporation
BMI	Body Mass Index
BP	Blood Pressure
HDL	High-Density Lipoprotein
LDL	Low-Density Lipoprotein
SCr	Serum Creatinine
AST	Aspartate Aminotransferase
ALT	Alanine Aminotransferase
GGT	γ-glutamyltransferase
eGFR	Estimated Glomerular Filtration Rate
CKD-EPI	Chronic Kidney Disease Epidemiology Collaboration
SD	Standard Deviations
IQR	Interquartile Ranges
CKD	Chronic Kidney Disease
SIR	Standardized Incidence Ratio
CI	Confidence Intervals
HR	Hazard Ratio
Ang II	Angiotensin II
ACE	Angiotensin-Converting Enzyme

## References

[1] Morgan E., Arnold M., Camargo M.C., Gini A., Kunzmann A.T., Matsuda T., Meheus F., Verhoeven R.H.A., Vignat J., Laversanne M. (2022). The Current and Future Incidence and Mortality of Gastric Cancer in 185 Countries, 2020–40: A Population-Based Modelling Study. eClinicalMedicine.

[2] Zhang T., Zhang Y., Leng X. (2024). Global, Regional, and National Trends in Gastric Cancer Burden: 1990–2021 and Projections to 2040. Front. Oncol..

[3] Mousavi S.E., Ilaghi M., Elahi Vahed I., Nejadghaderi S.A. (2025). Epidemiology and Socioeconomic Correlates of Gastric Cancer in Asia: Results from the GLOBOCAN 2020 Data and Projections from 2020 to 2040. Sci. Rep..

[4] Park E.H., Jung K.-W., Park N.J., Kang M.J., Yun E.H., Kim H.-J., Kim J.-E., Kong H.-J., Im J.-S., Seo H.G. (2024). Cancer Statistics in Korea: Incidence, Mortality, Survival, and Prevalence in 2021. Cancer Res. Treat..

[5] Suh Y., Lee J., Woo H., Shin D., Kong S., Lee H., Shin A., Yang H. (2020). National Cancer Screening Program for Gastric Cancer in Korea: Nationwide Treatment Benefit and Cost. Cancer.

[6] Kim Y.-I. (2024). Performance of the National Cancer Screening Program for Gastric Cancer in Korea. Korean J. Helicobacter Up. Gastrointest. Res..

[7] Brooks E.R., Siriruchatanon M., Prabhu V., Charytan D.M., Huang W.C., Chen Y., Kang S.K. (2024). Chronic Kidney Disease and Risk of Kidney or Urothelial Malignancy: Systematic Review and Meta-Analysis. Nephrol. Dial. Transplant..

[8] Lees J.S., Elyan B.M.P., Herrmann S.M., Lang N.N., Jones R.J., Mark P.B. (2023). The ‘Other’ Big Complication: How Chronic Kidney Disease Impacts on Cancer Risks and Outcomes. Nephrol. Dial. Transplant..

[9] Ahn S.Y., Choi Y.J., Han K., Ko G.J., Kwon Y.J., Park Y.-G. (2020). Dipstick Proteinuria and Cancer Incidence: A Nationwide Population-Based Study. J. Nephrol..

[10] Lee J., Lee J.S., Park S.-H., Shin S.A., Kim K. (2016). Cohort Profile: The National Health Insurance Service–National Sample Cohort (NHIS-NSC), South Korea. Int. J. Epidemiol..

[11] Huang M.-M., Guo A.-B., Sun J.-F., Chen X.-L., Yin Z.-Y. (2014). Angiotensin II Promotes the Progression of Human Gastric Cancer. Mol. Med. Rep..

[12] Kim S.T., Park K.H., Oh S.C., Seo J.H., Kim J.S., Shin S.W., Kim Y.H. (2012). How Does Inhibition of the Renin-Angiotensin System Affect the Prognosis of Advanced Gastric Cancer Patients Receiving Platinum-Based Chemotherapy?. Oncology.

[13] Park S., Lee S., Kim Y., Lee Y., Kang M.W., Han K., Han S.S., Lee H., Lee J.P., Joo K.W. (2019). Risk of Cancer in Pre-Dialysis Chronic Kidney Disease: A Nationwide Population-Based Study with a Matched Control Group. Kidney Res. Clin. Pract..

[14] Suzuki Y., Kaneko H., Okada A., Fujiu K., Michihata N., Jo T., Takeda N., Morita H., Kamiya K., Matsunaga A. (2022). Proteinuria and Risk for Heart Failure in 55,191 Patients Having History of Cancer. Am. J. Nephrol..

[15] Ager E.I., Neo J., Christophi C. (2008). The Renin–Angiotensin System and Malignancy. Carcinogenesis.

[16] Kwon S.K., Han J.-H., Kim H.-Y., Kang G., Kang M., Kim Y.J., Min J. (2019). The Incidences and Characteristics of Various Cancers in Patients on Dialysis: A Korean Nationwide Study. J. Korean Med. Sci..

[17] Paisley K.E., Beaman M., Tooke J.E., Mohamed-Ali V., Lowe G.D.O., Shore A.C. (2003). Endothelial Dysfunction and Inflammation in Asymptomatic Proteinuria. Kidney Int..

[18] Khan F.H., Dervan E., Bhattacharyya D.D., McAuliffe J.D., Miranda K.M., Glynn S.A. (2020). The Role of Nitric Oxide in Cancer: Master Regulator or Not?. Int. J. Mol. Sci..

[19] Ying L., Hofseth L.J. (2007). An Emerging Role for Endothelial Nitric Oxide Synthase in Chronic Inflammation and Cancer. Cancer Res..

[20] Borrego S., Vazquez A., Dasí F., Cerdá C., Iradi A., Tormos C., Sánchez J., Bagán L., Boix J., Zaragoza C. (2013). Oxidative Stress and DNA Damage in Human Gastric Carcinoma: 8-Oxo-7′8-Dihydro-2′-Deoxyguanosine (8-Oxo-dG) as a Possible Tumor Marker. Int. J. Mol. Sci..

[21] Juneja V.R., McGuire K.A., Manguso R.T., LaFleur M.W., Collins N., Haining W.N., Freeman G.J., Sharpe A.H. (2017). PD-L1 on Tumor Cells Is Sufficient for Immune Evasion in Immunogenic Tumors and Inhibits CD8 T Cell Cytotoxicity. J. Exp. Med..

[22] Srinivas U.S., Tan B.W.Q., Vellayappan B.A., Jeyasekharan A.D. (2019). ROS and the DNA Damage Response in Cancer. Redox Biol..

[23] Farinati F., Cardin R., Degan P., Rugge M., Di Mario F., Bonvicini P., Naccarato R. (1998). Oxidative DNA Damage Accumulation in Gastric Carcinogenesis. Gut.

[24] Jiang L., Hickman J.H., Wang S.-J., Gu W. (2015). Dynamic Roles of P53-Mediated Metabolic Activities in ROS-Induced Stress Responses. Cell Cycle.

[25] Kim Y.-I., Choi I.J. (2022). Current status of the gastric cancer screening program in Korea. J. Korean Med. Assoc..

[26] Choi K.S., Suh M. (2014). Screening for Gastric Cancer: The Usefulness of Endoscopy. Clin. Endosc..

[27] Al-Alawi A., Mulgrew A., Tench E., Ryan C.F. (2006). Prevalence, Risk Factors and Impact on Daytime Sleepiness and Hypertension of Periodic Leg Movements with Arousals in Patients with Obstructive Sleep Apnea. J. Clin. Sleep Med..

[28] Pedersen L.M., Milman N. (1996). Prevalence and Prognostic Significance of Proteinuria in Patients with Lung Cancer. Acta Oncol..

[29] Oh S.Y., Han K.-D., Ku G.Y., Kang W.-K. (2025). Association between Proteinuria Changes and Colorectal Cancer Incidence: Evidence from a Nationwide Cohort Study. BMC Gastroenterol..

[30] Mc Menamin Ú.C., Murray L.J., Cantwell M.M., Hughes C.M. (2012). Angiotensin-Converting Enzyme Inhibitors and Angiotensin Receptor Blockers in Cancer Progression and Survival: A Systematic Review. Cancer Causes Control.

[31] De Felice F., Malerba S., Nardone V., Salvestrini V., Calomino N., Testini M., Boccardi V., Desideri I., Gentili C., De Luca R. (2025). Progress and Challenges in Integrating Nutritional Care into Oncology Practice: Results from a National Survey on Behalf of the NutriOnc Research Group. Nutrients.

