# Supplementary material for: Prognostic Value of the Level of Urine Dipstick Proteinuria in Gastric Cancer in the Korean Population
Source: Cancers (Basel). 2025 Aug 23;17(17):2743. doi: 10.3390/cancers17172743 (PMC12427291; doi:10.3390/cancers17172743)
Supplement: Supplementary file 1 [file cancers-17-02743-s001.zip › cancers-3839563-supplementary.pdf]

Table S1. Comparison between participants with and without incident gastric cancer.

Characteristic	Without incident gastric cancer (N=219,007)	With incident gastric cancer (N=1,934)	*P*-value*
Age (years)	58.0 ± (8.7)	62.9 ± (9.5)	<0.001
Gender			<0.001
Male (%)	122,574 (56.0)	1,429(73.9)	
Female (%)	96,433 (44.0)	505 (26.1)	
BMI (kg/m^2)	24.0 ± (2.9)	23.9 ± (2.9)	0.240
Systolic BP (mmHg)	125.3 ± (15.2)	127.9 ± (15.6)	<0.001
Diastolic BP (mmHg)	77.7 ± (9.9)	78.6 ± (10.2)	<0.001
Total cholesterol (mg/dL)	200.1 ± (37.4)	196.7 ± (41.1)	<0.001
Triglyceride (mg/dL)	141.7 ± (93.8)	146.1 ± (95.3)	0.044
HDL-cholesterol (mg/dL)	55.4 ± (32.2)	54.7 ± (39.4)	0.460
LDL-cholesterol (mg/dL)	118.4 ± (39.0)	115.7 ± (39.3)	0.002
Fasting blood glucose (mg/dL)	100.8 ± (25.4)	103.4 ± (27.3)	<0.001
SCr (mg/dL)	1.15 ± (1.48)	1.21 ± (1.76)	0.145

eGFR (mL/min per 1.73m^2)	80.7 ± (20.2)	78.1 ± (19.4)	<0.001
Urine protein (%)			0.010
absence	97.1	96.3	
1+	1.9	2.0	
≥2+	1.0	1.7	
AST (U/L)	26.6 ± (16.4)	28.2 ± (17.5)	<0.001
ALT (U/L)	25.4 ± (19.3)	25.8 ± (18.4)	0.386
GGT (U/L)	39.0 ± (53.5)	47.2 ± (73.1)	<0.001
Smoking amount (pack-year)	7.7 ± (13.8)	11.5 ± (16.1)	<0.001
Alcohol intake (%)	14.3	20.4	<0.001
Physical activity (%)	16.9	18.3	0.112

Data are expressed as means (standard deviation) or percentages.

[*] *P*-value by t-test for continuous variables and Chi square test for categorical variables.
